# Put a RING on it: regulation and inhibition of RNF8 and RNF168 RING finger E3 ligases at DNA damage sites

**DOI:** 10.3389/fgene.2013.00128

**Published:** 2013-07-09

**Authors:** Cristina Bartocci, Eros Lazzerini Denchi

**Affiliations:** Laboratory of Chromosome Biology and Genomic Stability, Department of Molecular and Experimental Medicine, The Scripps Research InstituteLa Jolla, CA, USA

**Keywords:** RING E3 ligase, genomic stability, RNF8, RNF168, DNA damage response, ubiquitin, NHEJ, HR

## Abstract

RING (Really Interesting New Gene) domain-containing E3 ubiquitin ligases comprise a large family of enzymes that in combination with an E2 ubiquitin-conjugating enzyme, modify target proteins by attaching ubiquitin moieties. A number of RING E3s play an essential role in the cellular response to DNA damage highlighting a crucial contribution for ubiquitin-mediated signaling to the genome surveillance pathway. Among the RING E3s, RNF8 and RNF168 play a critical role in the response to double stranded breaks, one of the most deleterious types of DNA damage. These proteins act as positive regulators of the signaling cascade that initiates at DNA lesions. Inactivation of these enzymes is sufficient to severely impair the ability of cells to respond to DNA damage. Given their central role in the pathway, several layers of regulation act at this nodal signaling point. Here we will summarize current knowledge on the roles of RNF8 and RNF168 in maintaining genome integrity with particular emphasis on recent insights into the multiple layers of regulation that act on these enzymes to fine-tune the cellular response to DNA lesions.

## INTRODUCTION

Eukaryotic cells have evolved sophisticated mechanisms to detect and repair different types of DNA lesions, collectively referred to as the DNA damage response (DDR). In response to DNA lesions this pathway marks the damaged DNA, activates cell cycle checkpoints that halt cellular proliferation and activates the DNA damage repair machinery. Double strand DNA breaks are among the most deleterious types of DNA lesions and, strikingly, the presence of only a few DNA damage sites is sufficient to fully engage the DDR pathway resulting in a robust cell cycle inhibition (Bakkenist and Kastan, 2003). Several steps of regulation must ensure that the damage signal does not spread beyond the site of lesion and that its activation is reversed only upon completion of the DNA repair events. We now appreciate that an efficient response to DNA damage involves transcriptional regulation, micro-RNA biogenesis, detection of methylated histone tails, and multiple posttranslational modification events including phosphorylation, sumoylation, and ubiquitylation.

In this review, we will focus on the role of ubiquitin signaling in the response to double stranded breaks (DSBs). In recent years, an expanding view of ubiquitylation as a means to modulate the timing and efficiency of the repair process and to recruit factors to the sites of DNA lesions has emerged. Ubiquitin (Ub) is an essential 76-amino-acid protein conserved from yeast to humans. Ubiquitylation is a three-step enzymatic process through which ubiquitin becomes covalently attached to specific target proteins. First, ubiquitin is activated by an E1 activating enzyme via an ATP-dependent reaction to form an E1-Ub thioester bond. Then, the activated ubiquitin is transferred to an E2 conjugating enzyme. Finally, an E3 ligase catalyzes the transfer of ubiquitin to a target protein through formation of an isopeptide bond between the carboxyl-terminus of ubiquitin and a lysine (K) residue on the target protein (Ciechanover et al., 1982; Hershko et al., 1983). It is the E3 enzyme that confers the majority of the substrate specificity to the ubiquitylation cascade through recognition of a distinct set of target proteins.

The two major classes of E3 ligases are the RING (really interesting new gene)/Ubox domain-containing E3s and the HECT (homologous to E6-associated protein C-terminus) domain-containing E3s. While HECT E3s form an intermediate thioester bond with ubiquitin, RING E3s act as scaffolds, facilitating ubiquitylation by bringing the E2 and substrate close together.

RING finger ubiquitin ligases comprise one of the largest families of enzymes in human cells with more than 600 members (Deshaies and Joazeiro, 2009). They are the most abundant class of E3s and regulate many crucial cellular functions, such as cell cycle progression, DDR, DNA repair, cell signaling, and response to hypoxia (reviewed in Lipkowitz and Weissman, 2011). RING E3s catalyze target monoubiquitylation or polyubiquitylation assembled via different lysine residues of ubiquitin, which have a range of different biological effects, from proteasomal degradation (K48-linked polyubiquitylation) to regulation of DNA repair, receptor internalization, and gene silencing, among others (monoubiquitylation and/or K63-linked polyubiquitylation; Johnson, 2002; Sun and Chen, 2004).

Highlighting the relevance for ubiquitin signaling in organism homeostasis, mutations in RING E3s are frequently associated with human diseases such as BRCA1 (breast cancer type 1 susceptibility protein) mutations in patients with breast and ovarian cancer (Hashizume et al., 2001; Ruffner et al., 2001), and MDM2 (mouse double minute 2 homolog) amplification in several human cancers (Oliner et al., 1992; Wade et al., 2010).

Multiple RING E3s play a central role in various DDR pathways and are involved in either sensing or repairing DNA lesions, some of which are listed in **Table 1**.

**Table 1:** ING finger E3s involved in DDR pathways.

RING E3	Target	Ubiquitylation type	Function
RNF8	H2A/H2AX and other unknown	K63 chains	DSB signaling and repair
RNF168	H2A/H2AX	Mono on K13-15 of H2A/H2AX and K63 chains	DSB signaling and repair
BRCA1	Unknown	K6 and other unknown?	Promote HR
BMI1	H2A (H2AX?)	Mono on K119 of H2A	Gene silencing; DSB signaling?
RING1B	H2A (H2AX?)	Mono on K119 of H2A	Gene silencing; DSB signaling?
RAD18	PCNA	Mono on K164 of PCNA	PRR
FANCL	FANCD2/FANCI	Mono on K561 of FANCD2, on K523 of FANCI	ICL repair (FA pathway)

*Mono, monoubiquitylation; PRR, post-replication repair; ICL, inter-strand crosslink; FA, Fanconi anemia.*

In this review, we will focus primarily on RNF8 (RING finger 8) and RNF168 (RING finger 168), which are essential in the cellular response to DSBs and appear to form a key nodal point of regulation to modulate the DSB response and repair pathway.

We will also describe recently reported cross-talks between the RNF8/RNF168 pathway and other cellular components: (i) RING E3s of the polycomb group (PcG) complex PRC1 (polycomb repressive complex 1), which are emerging as potential novel players in the response to DSBs, where they appear to primarily contribute to the transcriptional silencing that occurs at sites of DNA lesions; (ii) proteins involved in small ubiquitin-like modifier (SUMO) signaling, whose role in DSB repair and response is becoming increasingly evident.

## RNF8/RNF168: MAJOR PLAYERS IN THE DETECTION OF DNA DOUBLE STRAND BREAKS

**FIGURE 1:**
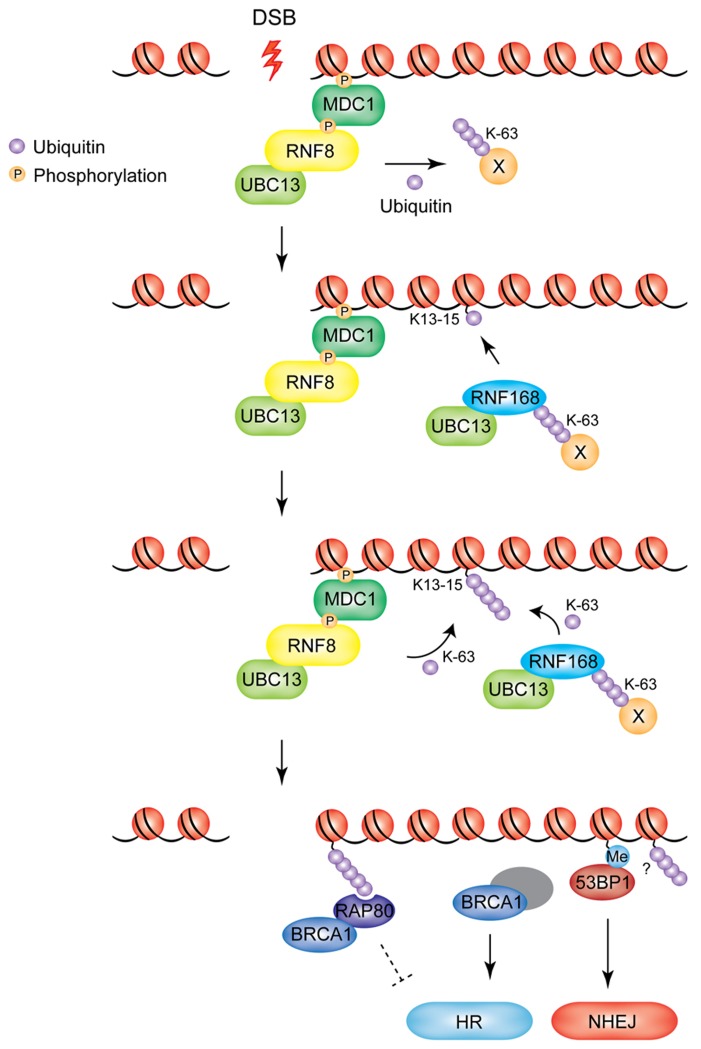
**Model of RNF8/RNF168-mediated ubiquitylation at DSBs.** RNF8 is recruited to DSBs through its interaction with MDC1. Chromatin-bound RNF8 cooperates with the E2 UBC13 to ubiquitylate an unknown non-nucleosomal target in the vicinity of the damaged chromatin (X). Ubiquitylated target-X is recognized by RNF168, which catalyzes monoubiquitylation of K13-15 on H2A-type histones. RNF8 and RNF168 work in concert to extend the ubiquitin chains on H2A-type histones. BRCA1 and 53BP1 are recruited as downstream effectors. BRCA1 accumulates at DSBs in an RNF8/RNF168-dependent manner, through RAP80, which binds to the K63-linked ubiquitin chains deposited by RNF8/RNF168. The RAP80–BRCA1 complex is thought to inhibit excessive HR, while BRCA1 in complex with several other DNA damage response proteins is known to primarily promote DNA repair by HR. 53BP1 accumulation at DSBs depends on RNF8/RNF168-mediated modifications to the chromatin surrounding the DNA lesion. 53BP1 promotes DNA repair by NHEJ (Me, methylation of H4K20).

The response to DSBs is initiated by the phosphatidylinositol 3-kinase-related kinase ATM (ataxia-telangiectasia mutated), which rapidly accumulates at DNA lesions in a MRN (MRE11/RAD50/NBS1)-dependent manner (Bekker-Jensen et al., 2006). ATM activation results in the phosphorylation of the histone variant H2AX on serine 139 (referred to as γ-H2AX). This marks the nucleosomes surrounding the DNA lesion and serves as an anchoring platform for the subsequent accumulation of downstream signaling proteins to DSB sites (Rogakou et al., 1998; Burma et al., 2001). MDC1 (mediator of DNA damage checkpoint 1) binds directly to γ-H2AX via its BRCT domains (Stucki et al., 2005). MDC1 recruitment results in amplification of the DNA damage signal by promoting further accumulation of the MRN complex at DSBs (Chapman and Jackson, 2008; Melander et al., 2008; Spycher et al., 2008). RNF8 and RNF168 are recruited at this step in the DDR pathway in an MDC1-dependent manner (**Figure 1**). RNF8 localizes to DSBs via its N-terminal forkhead-associated (FHA) domain, which interacts with the ATM-phosphorylated TQXF motifs on MDC1 (Huen et al., 2007; Kolas et al., 2007; Mailand et al., 2007). In concert with the E2 enzyme UBC13, the ligase activity of the C-terminal RNF8 RING finger domain is responsible for the recruitment of BRCA1 and 53BP1 (p53 binding protein 1; see below for more details; Wang and Elledge, 2007).

Historically, evidence has suggested that the targets of RNF8-UBC13 at DSBs are the histones H2A and H2AX, as they were shown to be ubiquitylated in response to DSBs in an RNF8-dependent manner (ubiquitylated H2A, uH2A; Huen et al., 2007; Mailand et al., 2007). Depletion of RNF8 leads to an impaired G2/M checkpoint at low doses of ionizing radiation (IR) and hyper-sensitivity to IR, indicating that the RNF8-dependent signaling is critical for the cellular response to DNA damage (Huen et al., 2007; Kolas et al., 2007; Mailand et al., 2007; Wang and Elledge, 2007).

RNF168 was identified in genome-wide screens as a factor necessary for IR-induced 53BP1 focus formation (Doil et al., 2009; Stewart et al., 2009). Significantly, mutations of RNF168 are associated with the RIDDLE syndrome (radiosensitivity immunodeficiency dysmorphic features and learning difficulties), which is characterized by cellular defects in repairing DSBs. Cells derived from RIDDLE patients fail to recruit 53BP1 and BRCA1 at irradiation-induced DNA damage sites (IRIFs) and can be complemented by exogenous RNF168 expression (Stewart et al., 2009). RNF168 is a RING-type ubiquitin ligase that also works with the UBC13 E2 enzyme. The RING domain of RNF168 is not required for recruitment at DNA lesions, which is instead dependent on its two ubiquitin-binding motifs MIU1 and MIU2 (motif interacting with ubiquitin; Doil et al., 2009; Pinato et al., 2009; Stewart et al., 2009; Panier et al., 2012). However, the catalytic activity of RNF168 is required for the accumulation of 53BP1 and BRCA1 at sites of damage. RNF168 activity is also required for the accumulation of IR-induced uH2A, an activity that can be recapitulated *in vitro* (Doil et al., 2009; Stewart et al., 2009). RNF168 recruitment at damage sites is RNF8-dependent, while RNF8 is recruited to DSBs in a RNF168-independent manner (Doil et al., 2009; Stewart et al., 2009). These data supported the widely accepted model for the sequential recruitment of RNF8 and RNF168 at sites of DNA damage, whereby RNF8 would initiate ubiquitin conjugation on histones H2A and H2AX. Subsequently, RNF168 would be recruited at damaged sites through the binding of its MIU domains to uH2A, resulting in further amplification of the ubiquitin signal via the formation of K63-linked polyubiquitin chains (Panier and Durocher, 2009). This model has been recently challenged by the discovery that RNF8 is inactive toward nucleosomal H2A (Mattiroli et al., 2012). This study provides evidence that the initial ubiquitylation of histone H2A is mediated by RNF168 at K13-15. The authors propose a model in which RNF8 is responsible for the ubiquitylation of other non-nucleosomal proteins localized at the DNA damage site, which would represent the docking site for RNF168. Thus, recruitment of RNF168 in this model is still dependent on RNF8 but does not involve ubiquitylation of nucleosomal H2A (or H2AX) as the priming step. RNF168 catalyses the monoubiquitylation of H2A and H2AX at K13-15. Subsequently, K63-linked ubiquitin chains can be conjugated by both E3 ligases in concert (**Figure 1**). This model is also in keeping with a recent report from the Durocher laboratory, which suggests the existence of two waves of RNF168 recruitment to DSBs: an initial transient recognition of RNF8-dependent ubiquitylation, followed by a more stable association to DSB-flanking chromatin promoted by RNF168 catalytic activity itself (Panier et al., 2012).

DNA damage-induced non-proteolytic polyubiquitin chains catalyzed by RNF8 and RNF168 in DSB-flanking chromatin serve as binding sites for the recruitment of the downstream effectors of the DDR pathway BRCA1 and 53BP1 (**Figure 1**, bottom panel) (Kolas et al., 2007; Mailand et al., 2007; Wang and Elledge, 2007; Doil et al., 2009). The relative dynamics with which these two components accumulate at break sites is extremely important to determine the choice of repair pathway the cell will take to ensure genome stability, underscoring the central role played by RNF8/RNF168 in orchestrating the DDR and repair pathways.

In mammalian cells, DSBs are predominantly repaired by the homologous recombination (HR) and the non-homologous end-joining (NHEJ) pathways. NHEJ is the primary repair mechanism during G0-, G1-, and early S-phases of the cell cycle (Delacote and Lopez, 2008). The NHEJ process ligates the broken DNA molecule back together and, due to the varying levels of end processing prior to end-joining, this pathway is often error-prone (reviewed in Lieber, 2008). Conversely, the HR pathway is an error-free repair process that utilizes the sister chromatid as a template to repair damaged DNA and is thus only active in the S/G2 phase of the cell cycle (Moynahan et al., 1999; Wyman and Kanaar, 2006). 53BP1 and BRCA1 have reciprocal roles in DSB repair: BRCA1 is required for efficient HR, while 53BP1 promotes NHEJ (see **Figure 1**; Nakamura et al., 2006; Xie et al., 2007; Difilippantonio et al., 2008; Dimitrova et al., 2008; Yun and Hiom, 2009). Several recent reports have shown how the antagonism between these two proteins is important for DSB repair pathway choice and consequent cell survival. A striking example of this antagonism is provided by cells that have impaired BRCA1 activity: inhibition of 53BP1 in this background is able to restore viability and suppress genomic instability associated with defective BRCA1, by allowing resection of DSBs and repair via the HR pathway to occur (Bouwman et al., 2010; Bunting et al., 2010). Below we will summarize current knowledge on how RNF8 and RNF168-dependent signaling recruits these important factors to sites of DSBs.

## MECHANISMS OF RNF8/RNF168-DEPENDENT RECRUITMENT OF DOWNSTREAM EFFECTOR PROTEINS

### 53BP1

Recruitment of 53BP1 to damaged chromatin was shown to occur via recognition of dimethylated histone H4 on K20 (H4K20me2) by its tandem Tudor domain (Sanders et al., 2004; Botuyan et al., 2006). 53BP1 does not contain any known ubiquitin-binding motifs and therefore the mechanism through which RNF8/RNF168 mediates its recruitment is still not fully understood. Recent findings from different groups point to a model in which RNF8/RNF168-dependent ubiquitylation at DSBs is necessary to remove factors that bind to H4K20me2 sites, thus unmasking the sites for 53BP1 binding. Meerang et al. (2011) demonstrated that RNF8 is responsible for the recruitment of the AAA ATPase p97/VCP to the sites of DNA damage via both K63- and K48-linked ubiquitin chains. p97/VCP was shown to be in turn responsible for the removal of the polycomb protein L3MBTL1 from H4K20me2 histones at damage sites (Acs et al., 2011). These findings suggest that removal of L3MBTL1 is necessary for 53BP1 binding to H4K20me2, although this hypothesis remains to be fully validated. In another study by Mallette et al. (2012), RNF8/RNF168-dependent unmasking of H4K20me2 sites was indeed found to be involved in 53BP1 recruitment to DSBs. In their work, the authors show that the Tudor domain-containing lysine demethylases JMJD2A and JMJD2B, which bind to H4K20me2, become polyubiquitylated and degraded in response to DNA damage and that this requires the E3 ligase activity of both RNF8 and RNF168. The combined knockdown of JMJD2A and JMJD2B significantly rescued the ability of RNF8- and RNF168-deficient cells to form 53BP1 foci upon DNA damage induction, indicating that the RNF8/RNF168-dependent degradation of the lysine demethylases controls the recruitment of 53BP1 at DNA damage sites. Although these findings indicate that removal of H4K20me2-binding proteins is certainly important for the recruitment of 53BP1 to DSBs, it is likely that other mechanisms exist which could explain the requirement for H2AK13-K15 ubiquitylation in this process (Mattiroli et al., 2012). An additional mechanism that may contribute to 53BP1 DSB recruitment is the DNA damage-induced chromatin relaxation that occurs at sites of DNA lesions, which may allow H4K20me2 to be more accessible for 53BP1 binding (Xu et al., 2010; Luijsterburg and van Attikum, 2012). A more detailed description of this process will be provided in Section “Chromatin Remodeling” of this review.

### BRCA1

BRCA1 is a RING E3 ubiquitin ligase and is recruited to DNA as a dimer with BARD1 (BRCA1-associated RING domain 1), which enhances BRCA1 RING domain ligase activity *in vitro*. BRCA1 has multiple roles in the DDR and repair pathways, underscored by its recruitment to damaged DNA in several different complexes. These complexes have functions in sensing DNA damage, controlling cell cycle checkpoints, and recruiting DNA repair enzymes to orchestrate the cross-talk of the DNA repair pathways with various cellular processes (Huen et al., 2010). It is generally accepted that the main function of BRCA1 in DNA repair is to promote HR (reviewed in Yun and Hiom, 2009; Caestecker and Van de Walle, 2013). At DSBs, BRCA1 is recruited to RNF8/RNF168-generated ubiquitin chains as part of the BRCA1-A complex [containing BRCA1, BARD1, RAP80 (receptor-associated protein 80), ABRAXAS, BRCC36, BRE, and NBA1] through RAP80, which binds to the K63-linked ubiquitin chains deposited by RNF8/RNF168 at DNA lesions via its two N-terminal UIMs (ubiquitin interacting motif; Kim et al., 2007; Sobhian et al., 2007; Wang and Elledge, 2007; Wang et al., 2007). The exact role of the BRCA1-A complex is not fully understood; although RAP80- and ABRAXAS-depleted cells display mild defects in HR (Wang et al., 2007), recent reports have shown that the BRCA1–RAP80 complex can inhibit HR by restricting end-resection (Coleman and Greenberg, 2011; Hu et al., 2011). Further work is required to establish the exact role of the different BRCA1 complexes in the repair of DSBs.

## MODULATION OF THE RNF8/168 PATHWAY

The choice of the DNA repair mechanism is crucial for cell survival after genotoxic insult. The relative accumulation at DSBs of 53BP1 and BRCA1, which determines this choice, is dependent on RNF8/RNF168, underscoring the necessity of a highly stringent regulation mechanism for these enzymes. In the following sections, we will illustrate the various means by which mammalian cells are able to achieve multiple layers of regulation in order to tightly control RNF8/RNF168 signaling, with particular focus on recent reports in the field. We will also describe how regulation of RNF8/RNF168 is exploited in different settings: by the telomere protection machinery to safeguard the integrity of chromosome ends, and by viruses during infection of mammalian cells.

### CHROMATIN REMODELING

Chromatin remodeling plays an important role in the DDR pathway to create a local chromatin environment that facilitates the assembly of checkpoint response and repair factors (reviewed in Luijsterburg and van Attikum, 2012; Soria et al., 2012). Indeed, several chromatin remodeling factors are recruited to sites of DNA damage and have been shown to modulate the activity of RNF8 and RNF168 at sites of damage.

**FIGURE 2:**
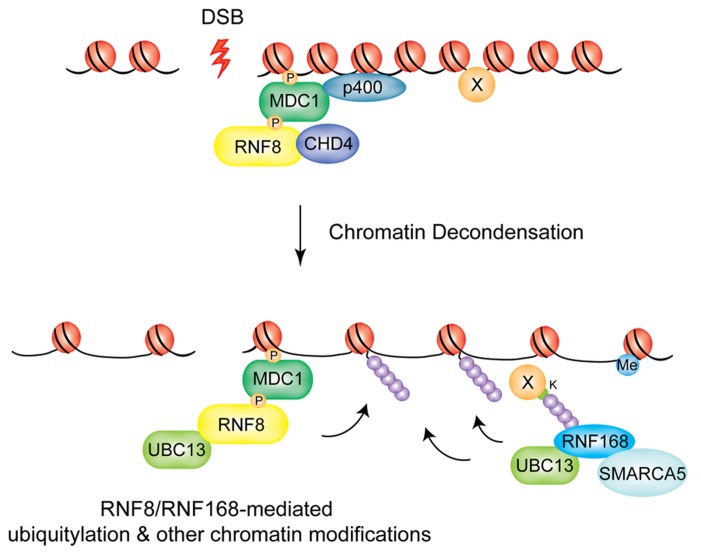
**Chromatin decondensation at DSBs.** The chromatin remodeling factors p400 and CHD4 mediate large-scale chromatin decondensation at DSB sites, promoting RNF8/RNF168-mediated ubiquitylation and other chromatin modifications. P400 is recruited by MDC1, CHD4 by RNF8. SMARCA5 is recruited through RNF168 and promotes its activity at DSBs.

p400, the catalytic subunit of the NuA4 histone acetyltransferase complex, is recruited at DSBs in a MDC1-dependent manner (Xu et al., 2010). Interestingly, in the absence of p400, while RNF8 is still recruited to DSBs, polyubiquitin chains are not formed at the sites of damage. As a consequence, BRCA1 and 53BP1 recruitment is impaired and p400-deficient cells show radiosensitivity and chromosomal aberrations. The mechanism through which this occurs is still unclear, but the authors speculate that chromatin relaxation may alter the nucleosome structure to expose previously buried histone domains, which can then be targeted by RNF8. In the light of the recent report from the Sixma group described above, one possibility is that p400-mediated chromatin relaxation is an important step to expose lysine residues on a so far unidentified RNF8 target (see model in **Figure 2**). This nucleosome destabilization was also suggested to contribute to exposure of methylated histone residues for binding of 53BP1.

A similar function seems to be played by CHD4 (chromodomain helicase DNA binding protein 4) of the NuRD histone deacetylase complex. CHD4 is recruited to laser-induced DNA damage sites in a RNF8-dependent manner, where it promotes efficient RNF8-mediated ubiquitin conjugation and recruitment of downstream factors RNF168 and BRCA1, presumably by favoring chromatin decondensation (Luijsterburg et al., 2012). Interestingly, RNF8 interaction with CHD4 involves the FHA domain of RNF8 while it does not require E3 catalytic activity.

Finally, SMARCA5, an ATPase contained in several distinct ISWI chromatin remodeling complexes has been shown to have a role in RNF8/RNF168-dependent DDR signaling (Smeenk et al., 2012). Smeenk and colleagues show that SMARCA5 is recruited to DSBs induced by laser micro-irradiation and its depletion increases IR-sensitivity and impairs both the HR and NHEJ repair pathways. SMARCA5 and RNF168 interact at DSBs in a mutually dependent fashion that requires the catalytic activity of PARP1. Surprisingly, SMARCA5-depleted cells do not display any defect in 53BP1 accumulation at IRIFs, even though ubiquitin conjugation and BRCA1 assembly were significantly reduced. This implies that either the threshold for ubiquitin levels required to promote BRCA1 or 53BP1 recruitment differs, or that alternative mechanisms of 53BP1 recruitment exist when the SMARCA5-RNF168 response is impaired. Further studies will be required to establish how SMARCA5 and the other chromatin remodeling enzymes described above remodel chromatin in the vicinity of DSBs to promote the RNF8/RNF168 signaling cascade, thereby promoting genomic stability in response to genotoxic insult.

Collectively, these data suggest a “chromatin remodeling-assisted ubiquitylation” activity for RNF8 and RNF168. In this model p400 and CHD4 are required to promote the initial ubiquitylation events at DNA damage sites that are needed to recruit RNF168, which, in turn, propagates the ubiquitylation signal and promotes accumulation of downstream effectors. In addition, SMARC5 plays a role at the level of RNF168 promoting its activity following the initial ubiquitylation event (see model in **Figure 2**).

### DE-UBIQUITYLATION ACTIVITIES AT SITES OF DNA DAMAGE

Ubiquitin-mediated signaling at sites of DNA damage can be attenuated or turned off by the action of de-ubiquitylating enzymes (DUBs; Sowa et al., 2009).

The human genome encodes nearly 100 DUBs that are divided into five classes based on their mechanism of catalysis (Nijman et al., 2005). The first four classes are papain-like cysteine proteases and include: the ubiquitin C-terminal hydrolases (UCHs), the ubiquitin-specific proteases (USPs), the ovarian tumor proteases (OTUs), and the Josephines. The fifth class is composed of the JAB1/MPN/MOV34 metalloenzymes (JAMMs), which are zinc-dependent metalloproteases.

**FIGURE 3:**
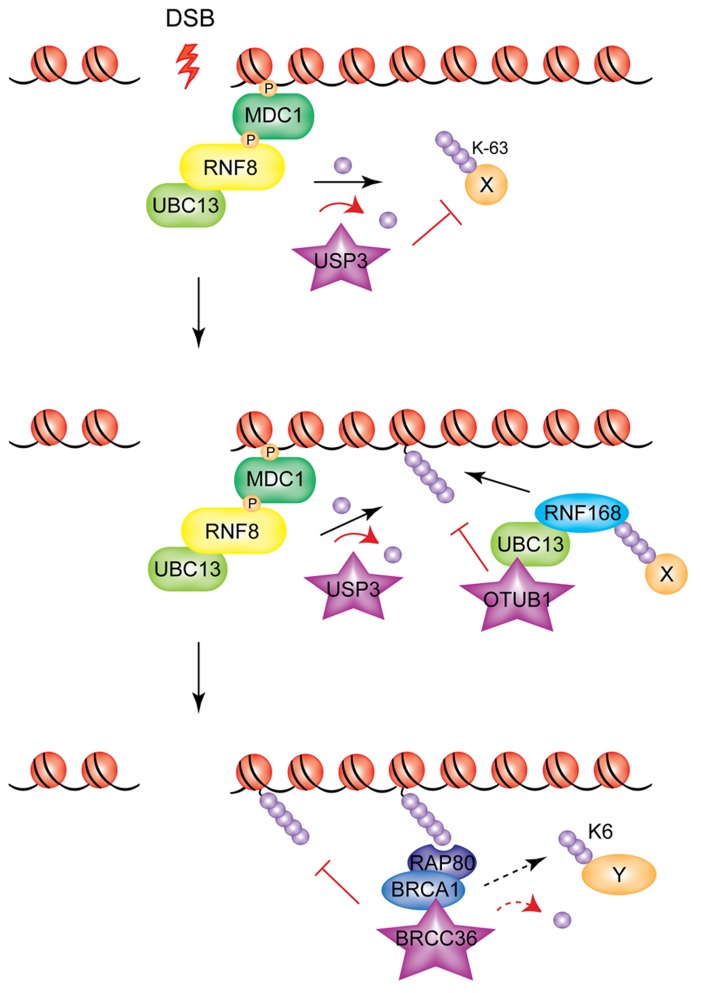
**Modulation of RNF8/RNF168 signaling through de-ubiquitylation.** Different DUBs act at different levels of the RNF8/RNF168 cascade to inhibit, modulate, or turn off the ubiquitin signal generated by the two E3s. USP3 de-ubiquitylates RNF8 targets; OTUB1 acts on substrates of RNF168, and BRCC36 acts downstream, severing ubiquitin chains generated presumably by both E3s (and possibly also on BRCA1 targets, indicated by the dashed arrow).

To date, four DUBs have been shown to counteract the action of RNF8/RNF168 on H2A/H2AX (see **Figure 3**): USP3, USP16 (ubiquitin-specific protease 3 and 16, respectively), BRCC36 (BRCA1/BRCA2-containing complex subunit 36) and OTUB1 (OUT domain ubiquitin aldehyde-binding 1).

USP3 has been shown to be a negative regulator of the RNF8/RNF168 pathway through its de-ubiquitylating activity (Nicassio et al., 2007). Overexpression of USP3, while having no effect on retention of RNF8 at DSBs, abolished the IR-induced focus formation of RNF168, RAP80, and 53BP1 (Doil et al., 2009), indicating that it de-ubiquitylates RNF8-targeted substrates.

USP16 is the major DUB for uH2A and acts as an antagonist of polycomb-dependent repression of gene expression (Joo et al., 2007). An ATM-dependent transcriptional silencing at DSBs has been reported, and seems to be at least partially mediated by the ubiquitin mark deposited by RNF8/RNF168 at H2A. This transcriptional repression at DSBs can be alleviated by inhibition of ATM (ATMi). USP16 depletion could prevent ATMi-mediated restoration of transcription, indicating that this DUB is responsible for de-ubiquitylation of uH2A at DSBs (Shanbhag et al., 2010). Whether the target for USP16-mediated de-ubiquitylation is uH2A deposited by RNF8/RNF168 or by other E3 ligases remains to be established (see “RING E3 Polycomb Proteins and the RNF8/RNF168 DSB Response Pathway” Section for more details).

The K63-specific BRCC36 is recruited to DSBs as part of the BRCA1-A complex (Shao et al., 2009). BRCC36-depletion increases DSB-associated ubiquitin and 53BP1 accumulation at DSBs. This increase is partially reversed following RNF8 depletion, consistent with a role for BRCC36 as negative regulator of the RNF8/RNF168 pathway via de-ubiquitylation of K63-linked ubiquitin chains. Although the reasons for the presence of a de-ubiquitylase function in the context of a complex that recognizes ubiquitin chains are not fully understood, it has recently been proposed that BRCC36 may function to remove K63-linked ubiquitin from one substrate, allowing BRCA1 to mediate the formation of K6-linked chains on the same or other substrates, thus providing a dynamic balance between formation and removal of ubiquitin signals at DSBs, potentially important for the subsequent steps of repair (Wu et al., 2012).

Finally, the DUB OTUB1 was also shown to be a negative regulator of the RNF8/RNF168 pathway, at the level of RNF168 (Nakada et al., 2010), albeit in a non-canonical fashion. Indeed, the ability of OTUB1 to inhibit RNF168-dependent ubiquitylation is independent of its catalytic activity, and is instead mediated by its binding to and inhibition of the E2 UBC13, which cooperates with RNF168, suggesting that E2 regulation could also represent a means to regulate the DDR pathway.

The concerted action of the DUBs described above has an important contribution in counteracting RNF8/RNF168-mediated ubiquitin signaling to prevent excessive spreading of damage signals at DSBs and to terminate the signal after repair processes have occurred (**Figure 3**). It is likely that additional DUBs acting on other RNF8/RNF168 targets and/or with different ubiquitin-conjugate specificity remain to be identified.

### REGULATION OF PROTEIN STABILITY

**FIGURE 4:**
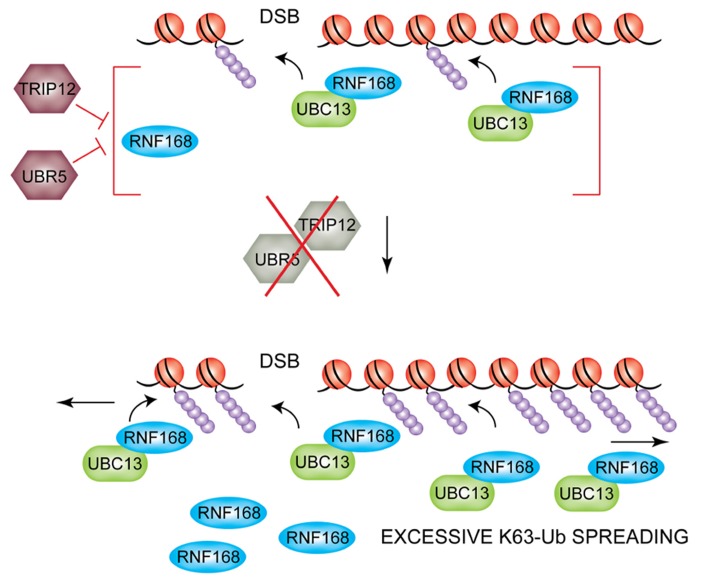
**TRIP12 and UBR5-mediated suppression of DSB-induced chromatin ubiquitylation.** The HECT E3 ligases TRIP12 and UBR5 control the nuclear pool of RNF168 (top). If TRIP12 and UBR5 are depleted, hyper-accumulation of RNF168 triggers excessive spreading of ubiquitylated H2A/H2AX far away from the initial site of DNA lesion (bottom).

An additional level of regulation that was recently uncovered acts at the level of RNF168 protein stability (Gudjonsson et al., 2012). Gudjonsson and colleagues show that RNF168 is a target for the HECT domain-containing E3s ligases, TRIP12 and UBR5, which reduce the pool of available nuclear RNF168 by targeting it for proteasomal-mediated degradation. Given the processive nature of RNF168 and its persistent self-recruitment to damaged DNA, this regulation is critical to limit the cellular levels of RNF168 and thus prevent excessive spreading of DNA damage-induced chromatin ubiquitylation and consequent hyper-accumulation of ubiquitin-mediated genome caretakers to undamaged regions of the genome (**Figure 4**). Indeed, in TRIP12/UBR5-depleted cells IR-induced 53BP1 foci are larger indicating hyper-accumulation and excessive spreading of these DDR factors along the chromatin. This has important consequences on DSB repair efficiency and consequent cell survival. Boosting chromatin ubiquitylation by inhibiting TRIP12/UBR5 enhances the repair efficiency of IR-induced DSBs by increasing rates of NHEJ, consistent with increased loading of 53BP1 on damaged chromatin. Interestingly, a more modest but nonetheless significant increase in HR rates could also be observed in the same experimental settings. The authors speculate that this could be due to hyper-accumulation of BRCA1 at sites of DNA lesions, which also is observed in TRIP12/UBR5-depleted cells and which could facilitate HR of unresolved DSBs close to the G2/M transition, when progressive chromatin condensation starts to displace NHEJ factors such as 53BP1. In addition to a faster and/or more efficient repair of DSBs, TRIP12/UBR5-depleted cells also displayed an improved short-term survival rate after irradiation, indicating that RNF168 overproducing cells could provide selective advantage to cancer cells that proliferate under increased genotoxic stress. Aberrations of both TRIP12 and UBR5 have been described in certain types of cancer (Clancy et al., 2003; O’Brien et al., 2008; Yoo et al., 2011), and elevated RNF168 protein levels and expanded 53BP1 foci were found in a subset of advanced human papillomavirus (HPV)-positive tumors (Gudjonsson et al., 2012), suggesting that the homeostasis of ubiquitin signaling achieved through regulation of RNF168 levels as illustrated here could be subverted during tumorigenesis.

Another HECT E3 ligase, HERC2, had been previously reported by the same group to have an opposite effect on RNF168 protein levels, by promoting RNF168 stability and therefore facilitating the assembly of repair factors to DSBs (Bekker-Jensen et al., 2010). In addition, the authors also showed that HERC2 was able to promote DSB signaling and repair by facilitating the assembly of RNF8 with its E2 partner UBC13, thus underscoring the importance of yet another E3 ligase, HERC2, in the RNF8/RNF168-dependent pathway in response to DSBs.

### DOWNSTREAM REGULATION BY RNF169 COMPETITIVE BINDING TO RNF8/RNF168 UBIQUITYLATED TARGETS

The RNF168 paralog RNF169 was recently identified as a novel RING E3 ligase with an unexpected negative role in the DNA damage-induced ubiquitin signaling response. RNF169 has a similar domain architecture to RNF168, including an N-terminal RING finger domain and UMI and MIU motifs and a C-terminal MIU motif (Panier et al., 2012; Poulsen et al., 2012). However, the nature of RNF169 recruitment to sites of damage is different to that observed for RNF168. Indeed, while RNF168 recruitment involves interaction with RNF8 targets through its N-terminal UBD region, the corresponding domain in RNF169 is dispensable for RNF169 localization. Instead, RNF169 accumulates at IRIFs in an RNF168-dependent manner by binding to RNF168-dependent ubiquitin products through its C-terminal MIU2. Functional studies demonstrated that while RNF169 is dispensable for ubiquitin-dependent protein assembly at DSB foci, its overexpression leads to impaired recruitment of 53BP1, BRCA1, RAP80, and RAD18 at these sites. Therefore, it was proposed that RNF169 competes with these DDR factors for binding to RNF168-dependent ubiquitin-modified chromatin (Panier et al., 2012; Poulsen et al., 2012). Indeed in a study on RNF169 the Mailand laboratory showed that overexpression of this E3 ligase stimulates HR efficiency while negatively impacting on the levels of NHEJ. By delaying and limiting the association of 53BP1 to DSBs while only having a mild effect on BRCA1 accumulation, the authors suggest that RNF169 might function to limit the use of NHEJ to repair the DNA lesions, thus channeling the repair toward the error-free HR pathway.

The data summarized above support a model in which the DNA damage-dependent ubiquitylation pathway is wired in a self-limiting circuit involving both positive and negative regulations, which allows histone ubiquitylation near the DNA lesions but at the same time prevents its excessive spreading to undamaged regions of the genome. Assembly and disassembly of this circuit allows for the timely accumulation of genome caretakers at sites of DNA damage to ensure a correct repair of the lesion.

### REGULATION OF RNF168 AT TELOMERES

**FIGURE 5:**
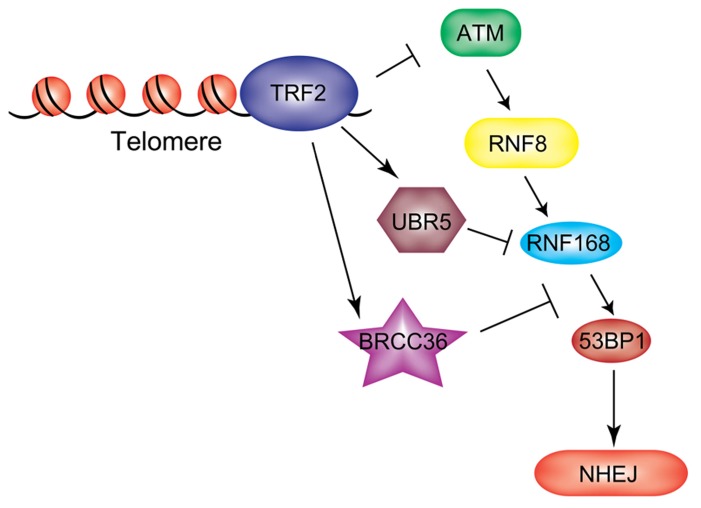
**RNF168 inhibition at chromosome ends.** TRF2 protects telomeres from unwanted activation of a DDR both by preventing upstream ATM activation and by inhibiting RNF168 ubiquitylation at these sites through recruitment of UBR5 and BRCC36.

Unprotected telomeres are perceived and processed by the cell as sites of DNA damage. This occurs upon attrition of telomeric DNA, or when specific components of shelterin are inhibited (Palm and de Lange, 2008). TRF2 (telomeric repeat binding factor 2) and POT1 (protection of telomeres 1) are involved in the suppression of the two major mammalian DDR pathways, mediated by ATM and ATR, respectively (Denchi and de Lange, 2007). Deletion of TRF2 from mouse cells leads to ATM activation and ATM-dependent formation of DNA damage foci at telomeres. Telomere dysfunction-induced foci (TIFs) contain the same repertoire of repair factors detected at DSBs, such as γH2AX, MDC1, and 53BP1 (Takai et al., 2003). Dysfunctional telomeres are processed by the NHEJ pathway resulting in end-to-end fusions (Celli et al., 2006). Recent findings from our lab uncovered a two-step mechanism of end protection mediated by distinct portions of TRF2. The first step requires the TRFH domain, which is involved in preventing the initial step of the DDR response, while the second step is mediated by a short amino-acid motif in the Hinge domain of TRF2, termed iDDR (inhibition of DDR) motif, that acts at the level of RNF168 (Okamoto et al., 2013). Expression of the iDDR region is sufficient to block the DDR signaling cascade at the level of RNF168 and prevent chromosome–chromosome fusions in TRF2-depleted cells. These data suggest that in mammalian cells repression of unwanted activation of the DDR at telomeres is achieved in part by inhibiting ubiquitin-dependent signaling at chromosome ends. The iDDR-dependent inhibition of RNF168 recruitment to dysfunctional telomeres requires the DUB enzyme BRCC36 and the ubiquitin ligase UBR5. This leads to a model in which RNF168 recruitment to telomeres is opposed by the concerted action of BRCC36 and UBR5 (**Figure 5**), underscoring again the importance of correctly regulating the RNF8/RNF168 pathway in order to ensure the maintenance of genomic stability.

### CONTROL OF RNF8/RNF168 DURING VIRAL INFECTION

The DDR is involved in the cellular response to viral infection: many viruses induce signaling through the same cellular cascade activated by the DDR, while some parts of the DDR itself are manipulated by virally encoded proteins in order to prevent detrimental outcomes for viral infection (Weitzman et al., 2010, 2011). Indeed the DDR may have adverse reactions on viral infection through checkpoint activation, recruitment of repressive factors and processing of viral DNA. Many viruses interact with the ubiquitin–proteasome system to block immune response and promote virus replication (Blanchette and Branton, 2009; Isaacson and Ploegh, 2009; Randow and Lehner, 2009; Viswanathan et al., 2010). E3 enzymes confer the majority of the substrate specificity to the ubiquitylation cascade; therefore, it is not surprising that this step is often targeted by viral proteins. One such example is the viral E3 ligase ICP0, encoded by the herpes simplex virus type 1 (HSV-1). ICP0 is a RING finger E3 that induces proteasomal degradation of several cellular proteins including the catalytic subunit of DNA-dependent protein kinase (DNA-PKcs), components of promyelocytic leukemia (PML) nuclear bodies, and centromeric proteins (Everett et al., 1998, 1999; Parkinson et al., 1999; Lomonte et al., 2001; Gu and Roizman, 2003; Lomonte and Morency, 2007). Work from the Weitzman laboratory has uncovered that ICP0 also targets RNF8 and RNF168, consequently blocking the DDR pathway at this level to prevent downstream repair proteins from accumulating at sites of cellular damage (Lilley et al., 2010). ICP0 expression promotes degradation of both RNF8 and RNF168 independently in a RING-dependent manner. RNF8 was shown to be targeted by ICP0 through the exploitation of a cellular phosphorylation-based strategy in a more recent study from the same laboratory (Chaurushiya et al., 2012). ICP0 is phosphorylated by the cellular kinase CK1 on T67, mimicking the phospho-sites induced on MDC1 during the DDR signaling. This phospho-site directly binds the RNF8 FHA domain, competing with the interaction with MDC1, and is responsible for RNF8 degradation.

Given that viruses have evolved to target key convergence points in cellular pathways, the data described above once again highlight the importance of the RNF8/RNF168 pathway as a nodal point in the DDR pathway that needs to be tightly regulated.

## RING E3 POLYCOMB PROTEINS AND THE RNF8/RNF168 DSB RESPONSE PATHWAY

**FIGURE 6:**
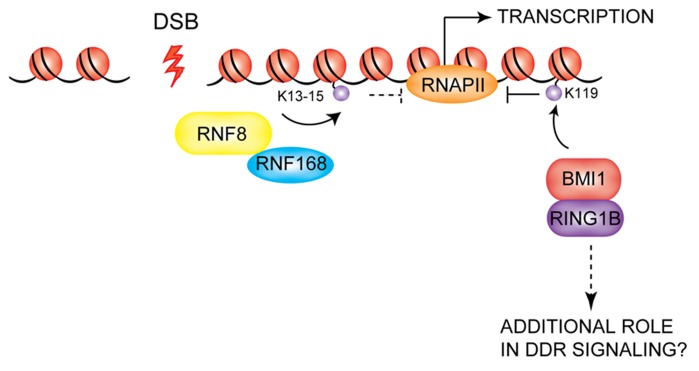
**Possible role of PcG proteins in the DDR.** BMI1/RING1B are recruited to DSBs, where they are thought to mediate the transcriptional silencing observed at DSBs via deposition of the K119 mark on H2A-type histones. RNF8/RNF168 also may contribute to gene repression at DSBs. BMI1/RING1B may also play additional roles in DDR signaling which remain to be uncovered.

Recent studies have uncovered a function for PcG proteins in the cellular response to DNA damage that may partially overlap with that of RNF8/RNF168. PcG proteins are chromatin-associated proteins that control a variety of cellular processes such as maintenance of cellular identity, proper embryonic development, and cell differentiation and proliferation (Pietersen and van Lohuizen, 2008; Bracken and Helin, 2009; Sauvageau and Sauvageau, 2010; Surface et al., 2010). Given the well-established role of PcG proteins in gene silencing, it is likely that PcGs contribute to the transcriptional repression observed at DSBs. Gene silencing at sites of DNA lesions was shown to be ATM-dependent and able to spread across kilobases of DNA *in cis* to the damage (Cuozzo et al., 2007; O’Hagan et al., 2008; Shanbhag et al., 2010). An important effector of this phenomenon is H2A ubiquitylation: the levels of uH2A at DSBs are strongly reduced upon ATM inhibition after DNA damage induction (while no effect is seen on K63-linked ubiquitin species at the same site) and this reduction correlates with a reversal of repression of transcription (Shanbhag et al., 2010). It has been demonstrated that expression of a mutant form of H2A that cannot be monoubiquitylated at K119 partially rescues transcription. A heterodimer of the E3 ligases BMI1 and RING1B of PRC1 catalyzes histone H2A monoubiquitylation at K119 (Wang et al., 2004; Cao et al., 2005), promoting gene silencing partly exerted by controlling the RNA polymerase II-mediated elongation phase of transcription (Stock et al., 2007). Thus, BMI1/RING1B may be the E3s responsible for the deposition of the H2AK119Ub repressive mark at sites of DNA lesions. Residues K13-15 of H2A/H2AX were recently identified as the target lysines for RNF8/RNF168 (Mattiroli et al., 2012), thus distinguishing the RNF8/RNF168 target from that of BMI1/RING1B (K119), suggesting that ubiquitylation of these two sites provides independent signals in the response to DSBs. In their study, Shanbhag and colleagues found that co-depletion of RNF8 and RNF168 also lead to a partial rescue of transcription levels at DSBs in cells treated with an ATM inhibitor after DNA damage induction. Whether RNF8/RNF168-dependent deposition of uH2A on K13-15 also directly plays a role in the induction of gene silencing at DSBs remains to be established (**Figure 6**). In this context, it will be important to elucidate the functional cross-talk between the RNF8/RNF168 ubiquitin pathway and the PcG proteins to determine if BMI1/RING1B also play a role in coordinating DSB signaling in addition to their hypothesized role in gene silencing at these sites. Several laboratories have recently shown that BMI1 and RING1B accumulate at sites of DSBs generated by IR or laser micro-irradiation, although there are some discrepancies between the different reports in terms of the effect of depletion of BMI1 on the recruitment of downstream DDR effectors and consequently on DNA repair (Chou et al., 2010; Facchino et al., 2010; Ismail et al., 2010; Chagraoui et al., 2011; Ginjala et al., 2011). Further studies will be required to elucidate the precise role for the PcG proteins in the DDR pathway.

It will also be interesting to determine whether DUBs have a role in regulating the activity of PcG E3 ligases in the context of the DSB response pathway, analogous to their contribution to the regulation RNF8/RNF168 as described above. The DUBs USP3 and USP11 are components of the PRC1 complex and are known to regulate BMI1 and MEL18 (another component of PRC1) turnover and abundance (Maertens et al., 2010). A novel PcG complex, polycomb repressive de-ubiquitinase (PR-DUB) has recently been described in *Drosophila* and is conserved in humans. It contains the BRCA1-complex associated protein 1 (BAP1), a tumor suppressor protein with DUB activity that is involved in de-ubiquitylation of H2AK119Ub (Ventii et al., 2008; Scheuermann et al., 2010). In addition, depletion of USP16, which acts as an antagonist of polycomb-dependent repression of gene expression (Joo et al., 2007), was shown to prevent the reversal of transcriptional silencing and decrease of uH2A levels observed at DSBs upon ATM inhibition or cessation of DNA damage. This indicates that USP16 is responsible for de-ubiquitylation of uH2A at DSBs (Shanbhag et al., 2010). Whether USP16 targets the products of RNF8/RNF168- and/or BMI1/RING1B-dependent ubiquitylation remains to be established.

## THE RNF8/RNF168 PATHWAY AND SUMO-DEPENDENT SIGNALING

Increasing evidence supports an important role for sumoylation in promoting the response to DSBs (reviewed in Bekker-Jensen and Mailand, 2011; Praefcke et al., 2012). Similarly to ubiquitylation, sumoylation is a process through which a SUMO moiety is covalently attached to a substrate protein. SUMO1, SUMO2/3, and the SUMO E3 ligases PIAS1 and PIAS4 accumulate at DSBs; PIAS1 and PIAS4 were shown to be responsible for sumoylation of 53BP1 as well as BRCA1 and their consequent accrual and/or retention at IRIFs (Galanty et al., 2009; Morris et al., 2009). Furthermore, a recent study from the Mailand group demonstrated that sumoylation of HERC2 and RNF168 by PIAS4 promotes ubiquitin-mediated protein assembly at DSB-surrounding chromatin, indicating that sumoylation is involved in regulation of the RNF8/RNF168 pathway (Danielsen et al., 2012). RNF8 or RNF168 depletion does not prevent PIAS1/4 accumulation at DSBs, but it does reduce the accrual of SUMO1 and SUMO2/3 to the damage sites. Thus the mechanism through which PIAS1 and PIAS4 are recruited to DSBs is independent of RNF8/RNF168, while presence of sumoylated protein species at damaged chromatin is downstream of RNF8/RNF168, likely because these ligases are in first instance required for recruitment of target proteins such as BRCA1 and 53BP1, which are subsequently sumoylated.

An additional link between the RNF8/RNF168 pathway and sumoylation is provided by the findings that the SUMO-targeted ubiquitin ligase (STUbL) RNF4, which is also recruited to DSBs, generates hybrid SUMO-ubiquitin chains at sites of DNA lesions which are critical for the recruitment of RAP80 and BRCA1 to the damaged chromatin and for consequent DNA repair (Prudden et al., 2007; Galanty et al., 2012; Guzzo et al., 2012). RNF4 is not required for RNF8/RNF168 recruitment to DSBs, but its depletion leads to delayed clearance of RNF8, RNF168, and 53BP1 from DSB foci and, strikingly, to a significant decrease in K63-linked ubiquitin chains that are deposited at DSBs (Yin et al., 2012). One possibility is that the mixed SUMO-ubiquitin chains generated by RNF4 are required to amplify the ubiquitin signal deposited by RNF8/RNF168. Further studies are required to understand the exact role of RNF4 at DSBs and how the complex interplay between RNF8/RNF168-dependent ubiquitin signaling and SUMO signaling at sites of DNA lesions contributes to DNA repair and cell survival.

## CONCLUDING REMARKS AND FUTURE PERSPECTIVES

The DDR and repair pathways are of vital importance to correct damages in the genome and ensure genomic stability. A wide variety of human genome instability syndromes exist whose underlying cause is the inactivation of DDR and repair genes (reviewed in Kerzendorfer and O’Driscoll, 2009). A paradigm for such syndromes is ataxia-telangiectasia (A-T), caused by a bi-allelic mutation in ATM (Savitsky et al., 1995). A-T symptoms are progressive neurodegeneration, immune dysfunction, hyper-sensitivity to IR, and marked cancer predisposition (Lavin and Shiloh, 1997). Inaccurate repair of DSBs can lead to chromosomal rearrangements that promote tumorigenesis (Jeggo and Lobrich, 2007). The present review focused on the multiplicity of regulation mechanisms that have evolved to tightly control one important nodule of the DDR signaling cascade, the RNF8/RNF168 pathway. Mutations of RNF168 are associated with the RIDDLE syndrome, which is characterized by defects in repairing DSBs (Stewart et al., 2009). It will be important to determine if RNF8/RNF168 activity opposes tumor formation and whether the DDR ubiquitylation pathway could represent a novel therapeutic target for cancer treatment. The multiple regulations of the RNF8/RNF168 pathway represent potential therapeutic targets, for example, in situations in which compromised ubiquitylation activity has a radioprotective function in cancer cells. In these cases the inhibition of specific DUBs may represent a viable therapeutic strategy.

Polycomb group-mediated repression of tumor suppressor genes is causally linked to cancer development (Bracken and Helin, 2009). The recent finding that PcG proteins may be involved in the response to DSBs provides an incentive to develop small-molecule inhibitors of PcG in order to treat cancer in combination with standard treatment strategies such as chemotherapeutics or radiotherapy, which induce DNA damage.

## Conflict of Interest Statement

The authors declare that the research was conducted in the absence of any commercial or financial relationships that could be construed as a potential conflict of interest.
